# Natural gums as sustained release carriers: development of gastroretentive drug delivery system of ziprasidone HCl

**DOI:** 10.1186/2008-2231-20-58

**Published:** 2012-10-17

**Authors:** Rajamma AJ, Yogesha HN, Sateesha SB

**Affiliations:** 1 Department of Pharmacognosy, KLE University’s College of Pharmacy, Bangalore, 560010, India; 2 Department of Pharmaceutics, Acharya & BM Reddy College of Pharmacy, Soladevanahally Hesaraghatta road, Bangalore, 560090, India

**Keywords:** Okra gum, Locust bean gum, Ziprasidone HCl, Gastro retentive tablet, Simplex lattice design, *In vivo* floatability

## Abstract

**Background:**

Objective of this study is to show the potential use of natural gums in the development of drug delivery systems. Therefore in this work gastro retentive tablet formulations of ziprasidone HCl were developed using simplex lattice design considering concentration of okra gum, locust bean gum and HPMC K4M as independent variables. A response surface plot and multiple regression equations were used to evaluate the effect of independent variables on hardness, f_lag_ time, floating time and drug release for 1 h, 2 h, and 8 h and for 24 h. A checkpoint batch was also prepared by considering the constraints and desirability of optimized formulation to improve its *in vitro* performance. Significance of result was analyzed using ANOVA and *p < 0.05* was considered statistically significant.

**Results:**

Formulation chiefly contains locust bean gum found to be favorable for hardness and floatability but combined effect of three variables was responsible for the sustained release of drug. The *in vitro* drug release data of check point batch (F8) was found to be sustained well compared to the most satisfactory formulation (F7) of 7 runs. The ‘n’ value was found to be between 0.5 and 1 suggesting that release of drug follows anomalous (non-fickian) diffusion mechanism indicating both diffusion and erosion mechanism from these natural gums. Predicted results were almost similar to the observed experimental values indicating the accuracy of the design. *In vivo* floatability test indicated non adherence to the gastric mucosa and tablets remain buoyant for more than 24 h.

**Conclusions:**

Study showed these eco-friendly natural gums can be considered as promising SR polymers.

## Introduction

The use of naturally occurring hydrophilic biocompatible polymeric materials has been focused in recent research activity in the design of oral controlled release dosage forms [1]. Natural gums are among the most popular hydrophilic polymers because of their cost-effectiveness and regulatory acceptance [2,3]. The use of naturally occurring plant-based pharmaceutical excipients has become very important in the development of controlled release dosage forms, because of their ability to produce a wide range of material based on their properties and molecular weight [4]. Plant based materials can be modified to meet the requirements of drug delivery systems and thus can compete with the synthetic excipients available in the market [5].

Okra gum and locust bean gum are water soluble thickening agents which have not been much studied for their pharmaceutical applications [6]. Okra gum, obtained from the fruits of *Hibiscus esculentus* L. (Moench), Malvaceae, is a polysaccharide consisting of D-galactose, L-rhamnose and L-galacturonic acid [7]. Locust bean gum (LBG) is a neutral plant galactomannan extracted from the seed (kernels) of the carob tree *Ceratonia siliqua* L. fabaceae [8]. The okra gum and LBG shows a synergistic gelation in acidic pH [9,10] and in combination with HPMC K4M forms an original gelation which has an excellent buoyancy and useful for oral gastro retentive formulations.

Ziprasidone HCl is an antipsychotic agent used in the treatment of schizophrenia [11]. The systemic bioavailability of ziprasidone administered intramuscularly is 100%, or 60%, administered orally with food. Drug reaches peak plasma concentration in 6 to 8 h after oral administration with an elimination half life of 7 h. This drug is more soluble in acidic pH and its solubility decreases with increasing pH owing to its pKa (~6) value [12]. The beneficial delivery system would be gastroretentive drug delivery systems which remain in the gastric region for several hours and significantly prolong the gastric residence time of drugs [13]. Hence, the goal has been set to evaluate the potential of Okra gum and LBG in combination with HPMC K4 for gastro retentive drug delivery system of ziprasidone HCl using simplex lattice design (SLD).

## Materials and methods

Ziprasidone HCl (Sanofi Aventis Pharma, Ltd, India) was received as a gift sample. HPMC K4M, okra gum (okra seeds, market), locust bean gum (Sigma Aldrich, Germany), and polyvinyl pyrollidone (Sisco research laboratories Pvt. Ltd) were purchased. All other chemicals used in the study were of analytical grade. Stat-ease Design-Expert® software was used to design the formulation.

### Okra gum (pod mucilage)

The fresh *A. esculentus* fruits were collected and washed with water. The fruits were crushed and soaked in water for 5–6 h, boiled for 30 min and left to stand for 1 h to allow complete release of the mucilage. The mucilage was separated using a multi layer muslin cloth and was precipitated by adding acetone (three times the volume of filtrate). The precipitate obtained was collected, dried in an oven at 40°C, and passed through a sieve #80 to obtain discrete powder [14].

### Simplex lattice design

**Figure 1:**
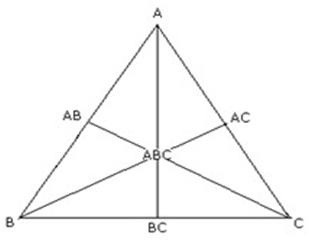
Equilateral triangle representing simplex lattice design for 3 components (A, B and C).

A simplex lattice design was adopted to optimize the formulation variables of gastro retentive drug delivery system of ziprasidone HCl [15]. The simplex lattice design for a 3-component system is represented by an equilateral triangle in 2-dimensional space (Figure 1). In this design, 3 factors were evaluated by changing their concentrations simultaneously and keeping their total concentration constant. Seven batches (F1-F7) of tablet formulations were prepared, one at each vertex (A, B, C), one at the halfway point between vertices (AB, BC, AC), and one at the center point (ABC). Each vertex represents a formulation containing the maximum amount of 1 component, with the other 2 components at a minimum level. The halfway point between the 2 vertices represents a formulation containing the average of the minimum and maximum amounts of the 2 ingredients. The center point represents a formulation containing one third of each ingredient.

Concentrations of HPMC K4M (A), okra gum (B) and LBG (C) were selected as independent variables. Hardness (kg/cm^2^), floating lag time (f_lag_ time, sec), drug release for 1 h (%), drug release for 2 h (%), drug release for 8h (%) and drug release for 24 h (%) were taken as response values (Dependent variables). The response values obtained were analyzed using multiple regression analysis to find out their relationship with the factors used.

### Formulation of gastroretentive matrix tablets

Weighed quantity of drug, polymers, effervescent combination and diluent (Table 1) were passed through sieve ^#^80, mixed and triturated in a mortar for a period of 10min to obtain uniform mixture. Powder was lubricated with magnesium stearate and talcum powder for 3min. Lubricated powder mass was compressed with 10-station Rimek Minipress RSB-1 tablet punching machine using 8 mm concave punches. The dimensional specifications were measured using thickness gauge (Okimoto); weight variation test was conducted as per pharmacopoeia of India specifications. Hardness of the tablet was measured using Pfizer type hardness tester.

**Table 1:** Formulations of ziprasidone HCl according to simplex lattice design

**Ingredients**	Formulation code*							
	**F1**	**F2**	**F3**	**F4**	**F5**	**F6**	**F7**	**F8**
Ziprasidone HCl	20	20	20	20	20	20	20	20
HPMC K4M	33.3	100	-	-	16.7	16.7	66.7	74.6
Okra gum	33.3	-	-	100	16.7	66.7	16.7	15.3
Locust bean gum	33.3	-	100	-	66.7	16.7	16.7	12.4
Sodium bicarbonate	40	40	40	40	40	40	40	40
Tartaric acid	10	10	10	10	10	10	10	10
Poly vinyl pyrrolidone	15	15	15	15	15	15	15	15
Magnesium stearate	1	1	1	1	1	1	1	1
Talc	1	1	1	1	1	1	1	1
Lactose Q.S	200	200	200	200	200	200	200	200

*All values are in mg.

### Drug content estimation

Standard calibration curve of ziprasidone HCl was constructed using UV-Visible spectrophotometer (Shimadzu-1700, Kyoto, Japan). Drug solution was prepared in methanol at the concentration range of 10 μg/mL to 50 μg/mL, sonicated, filtered using 0.45 μ (Millipore) membrane filter. The drug content of standard drug solution and tablet formulation was measured at 318 nm against methanol as a blank solution [16]. This method was found to have good repeatability, reproducibility and relative standard deviation (RSD) was not more than 2%. The working curve equation for ziprasidone HCl was y=0.011x with correlation coefficient value, r^2^ = 0.999.

### *In vitro* floatability

An *in vitro* floatability [17] of the formulation was determined by placing weighed tablet matrices in the USP dissolution testing apparatus II, in 900 ml of simulated gastric fluid (0.1N HCl, 0.2% NaCl) enzyme free at 37±0.5°C, rotated at 75 rpm.

The time required for the tablet to rise to the surface and float was determined as f_lag_ time. Floating time was the time, during which the tablet floats (including f_lag_ time) in simulated gastric fluid dissolution medium [18].

### Swelling index

The extent of swelling was measured in terms of percent weight gain by the tablet [19]. Each tablet formulation was kept in a beaker containing 100 mL of simulated gastric fluid; the tablet was withdrawn, blotted with tissue paper and reweighed. Then for every 1 h, weights of the tablets were noted and the process was continuous till the end of 6 h. The percentage weight gain by the tablet was calculated using the formula

$$\text{SI}\;=\;\left\{\left(M\text{t}\;-\;Mo\right)/Mo\right\}\;\times \;100\text{,}$$

where, SI is swelling index, *M*t is the weight of tablet at time “*t*”, and *M*o is the weight of tablet at time “*t*”=0.

### Dissolution studies

The release rate of ziprasidone HCl from floating matrix tablets were determined using USP XXIV dissolution apparatus (TDT-08T, Electrolab) Type-II (paddle) method for 24 h. Study was carried out using 900 ml of simulated gastric fluid (0.1 N HCl, 0.2% NaCl) enzyme free, at 37±0.5°C at 75 rpm. Aliquot volume of 5 ml was withdrawn from the dissolution apparatus hourly for 24 h and the samples were replaced with fresh prewarmed dissolution medium. The withdrawn samples were suitably diluted with methanol, filtered and drug content was determined using UV-spectrophotometer.

### Kinetic modeling on drug release profile

The dissolution profile of most satisfactory formulation of 7 runs and a check point batch (F8) were evaluated using mathematical models to describe the kinetics of the drug-release. The kinetics of drug release was evaluated for Higuchi, Korsmeyer-peppas, first order and zero order models to check the phenomena controlling the drug release from tablets [20,21]. The goodness of fit was evaluated using the correlation coefficient values (r^2^).

### Statistical analysis

The statistical assessment of simplex lattice design responses were performed using ANOVA and by applying the Student-t test. Model terms are significant if the calculated ‘t’ value is less than the critical value of ‘t’ (0.05).

### *In vivo* floatability

The *in vivo* floatability of F8 formulation loaded with barium sulphate was investigated by radiographic images (X-ray photographs) of rabbit’s stomach for specific period of time. Healthy rabbit weighing approximately 2.3 Kg was used to assess *in vivo* floating behaviour. The animal was fasted for 12 h and X-ray photograph was taken to ensure absence of radio opaque material in the stomach. The rabbit were made to swallow barium sulphate loaded tablet formulation with 30 ml of water. During the experiment rabbit were not allowed to eat but water was provided. At predetermined time intervals the radiograph of abdomen was taken to locate the formulation [22].

The preclinical study protocol was approved by Institutional Animal Ethical Committee, (Proposal No. IAEC/NCP/56/10) Nargund College of Pharmacy (NCP), Bangalore, Karnataka, India. Experiments were conducted according to the guidelines of committee for the “Purpose of Control and Supervision of Experiment on Animals” (CPCSEA).

## Results and discussion

### Formulation

The drug release characteristics were varied according to the types and proportion of matrix forming polymers in the formulation. HPMC K4M was selected as a hydrophilic matrixing agent [23]. LBG and Okra gum were considered as gelling agents they impart sufficient integrity to the tablets and works as release modifiers. Okra gum is insoluble in gastric pH but enormously swells which helps in retarding the drug release. Sodium bicarbonate generates CO_2_ gas in the presence of tartaric acid upon contact with dissolution medium. The gas generated is trapped and protected within the gel (formed by hydration of HPMC K4 M), thus decreasing the density of the tablet [24]. As the density of the tablet falls below 1 (density of water), the tablet becomes buoyant.

### Simplex lattice design

The general equation for the response based SLD for three components system consisting terms for pure component and mixtures of component [25].

(1) $$\text{R =}\;{\text{B}}_{0}\;+\;{\text{b}}_{1}\;\text{A +}\;{\text{b}}_{2}\;\text{B +}\;{\text{b}}_{3}\;\text{C}$$

where, R is the response variable and A, B and C are the proportions of formulation components. b_0_ is the arithmetic mean response of the 7 runs and b_1_, b_2_ and b_3_ are estimated coefficient for the factor A, B and C respectively. The coefficients can be calculated from the responses of ‘*R’* using a multiple regression equation**.** The fitted equations relating the hardness, f_lag_ time, and drug release for 1h, drug release for 2 h, drug release for 8h and drug release for 24 h to the transformed factor were used to draw conclusions after considering the magnitude of coefficient and the mathematical sign it carries (i.e., positive or negative).

### Effect of independent variables on hardness

(2) $$\text{R}1\;\left(\text{Hardness}\right)\;=\;5.44^{*}\text{A +}\;3.54^{*}\text{B +}\;5.90^{*}\text{C}$$

**Figure 2:**
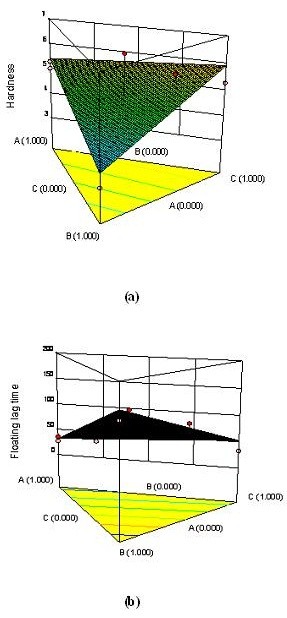
**Response surface plot showing the effect of concentration of HPMC K4M, okra gum and LBG on hardness (kg/cm**^**2**^**) and (b) floating lag time (sec).**

Although the statistical results infers {‘*F’* value of 3.04 and ‘*p’* value of 0.1370 (< 0.05)} the linear model equation is not significant for hardness, the values of regression coefficient infers, the concentration of HPMC K4M (A) and LBG (C) has equally contributed for the hardness (Figure 2a). Because HPMC K4M (A) and LBG (C) has sufficient cohesiveness and fibrous integrity makes them to undergo binding and contributed for hardness [26].

### Effect of independent variables on f_lag_ time

(3) $$\text{R}2\;{(\text{f}}_{\text{lag}}\;\text{time})\;=\;32.58^{*}\text{A +}\;54\text{.92*B +}\;52\text{.51*C}$$

The linear equation for f_lag_ time indicates that the factor ‘A’ has more significant effect on f_lag_ time than ‘B’ and ‘C’ (Figure 2b). This is further evident with the model terms for f_lag_ time being significant with ‘*F’* value of 16.63 and ‘*p’* value of 0.0062 (< 0.05) on a linear model. Floating lag time was found to increase at higher level of okra gum and decreases as the level of HPMC K4M increases. This is due to high swelling property of the later. Hence, a higher proportion of HPMC K4M is important in the formulation to decrease the f_lag_ time. This is also evident from the results of swelling index determination (221.95 to 257.15 (%) for F1 to F7 at the end of 6 h). Swelling index increases with increase in concentration of HPMC K4M signifying its importance for decrease in f_lag_ time [27].

### Effect of independent variables on drug release

The magnitude of coefficients observed for 1, 2, 8 and 24 h release obtained from the results of multiple linear regression analysis is expressed in equations 4, 5, 6 and 7 respectively. The release rate and percentage drug release for the 7 batches (F1 to F7) showed a wide variation (i.e., 80 to 95%) as shown in Table 2. Formulation F2 prepared using only HPMC K4M, exhausted before 8h and fails to sustain the drug release till 24 h. This highest value of percentage release observed in initial hours is due to low value of both the independent variables (B and C), thus weakening the gel strength.

**Table 2:** Characterization of ziprasidone HCl gastroretentive formulation

**Formulation code**	**Responses (Dependent variables)**					
	**Hardness (kg/cm**^**2**^**)**	**f**_**lag **_**time (sec)**	**Drug release for 1 h (%)**	**Drug release for 2 h (%)**	**Drug release for 8 h (%)**	**Drug release for 24 h (%)**
F1	6.52± 0.33	92.0± 5.19	4.86± 0.63	9.29± 0.81	27.39± 0.63	80.50± 0.83
F2	5.04±0.55	27.0± 5.29	11.31± 0.47	21.05± 0.23	89.39± 0.46	-
F3	5.24± 0.45	32.66± 3.51	4.39± 0.39	8.02± 0.40	25.69± 0.40	82.36± 0.39
F4	3.00± 0.37	137.0± 27.87	10.13± 0.60	14.79± 0.59	31.67± 0.45	85.45± 0.60
F5	5.66± 0.42	88.66± 14.57	4.93± 0.82	6.60± 0.40	30.55± 1.89	87.75± 1.03
F6	4.24± 0.38	136.0± 16.09	4.83± 0.80	6.56± 0.34	31.05± 0.44	82.26±0.41
F7	5.16±0.29	43.33± 13.79	5.61± 1.00	8.52± 0.61	31.31± 0.61	95.98± 0.47
F8	5.68± 0.52	37.33± 4.16	8.70± 0.41	14.83± 0.63	47.53± 0.87	97.58± 0.63

^a^All values are mean of 3 readings ± SD.

#### Drug release for 1h and 2h

(4) $$\begin{array}{l} \text{R}3\;\left(\text{Drug}\;\text{relese for}\;1\;\text{h}\right)\\ \;=\;9.75^{*}\text{A +}\;7.52^{*}\text{B +}\;2.97^{*}\text{C} \end{array}$$

(5) $$\begin{array}{l} \text{R}4\;\left(\text{Drug}\;\text{release for}\;2\;\text{h}\right)\\ \;=\;16.80^{*}\text{A +}\;10.47^{*}\text{B +}\;5.07^{*}\text{C} \end{array}$$

The equations 4 and 5 infer that the ‘A’ has more favorable effect on increase in drug release and the factor ‘B’ and ‘C’ in retarding drug release for 1 and 2 h. Although, the model terms are not significant {‘*p’* value of 0.1218 and 0.1315 (<0.05) for 1 h and 2 h drug release} it is understood that the water solubility of HPMC helps in increasing drug release and the water insolubility but the swellability of LBG and okra gum is responsible for it [28]. Optimum concentration of HPMC must be there in the formulation for immediate release of drug at initial hours.

#### Drug release for 8h and 24h

**Figure 3:**
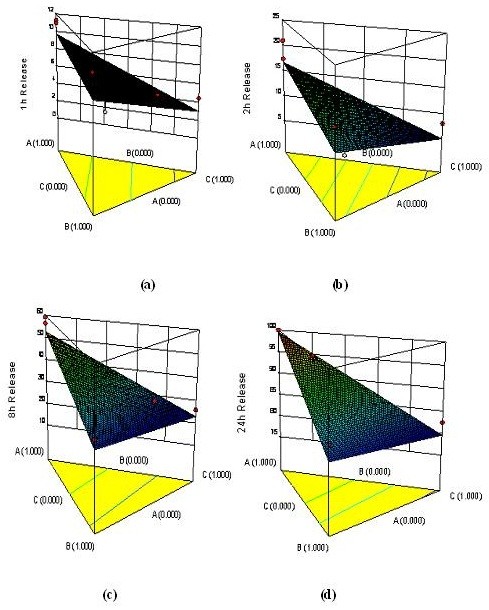
Response surface plot showing the effect of concentration of HPMC K4M, okra gum and LBG on % drug release for (a) 1 h, (b) 2 h, (c) 8 h and (d) 24 h respectively.

Concentration of HPMC K4M has important role in enhancing the drug release for 8 and 24 h and reverse is true with concentration of okra gum and LBG. As the concentration of okra gum and LBG increases, it causes an increase in viscosity of the swollen gel matrix, which decreases the water diffusion in to the core layer. Decrease in hydration of matrix contributes more hindrance for drug diffusion and consequently decrease in release rate [29]. This can be further elucidated with the help of response surface plot (Figure 3).

(6) $$\begin{array}{l} \text{R}5\;\left(\text{Drug}\;\text{release for}\;8\;\text{h}\right)\\ \;=\;52.87^{*}\text{A +}\;27.64^{*}\text{B +}\;22.86^{*}\text{C} \end{array}$$

(7) $$\begin{array}{l} \text{R}6\;\left(\text{Drug}\;\text{release for}\;24\;\text{h}\right)\\ \;=\;99.53^{*}\text{A +}\;82.02^{*}\text{B}\;+79.55^{*}\text{C} \end{array}$$

The model terms for *R5* (8 h release) and *R6* (24 h release) were found to be significant with an ‘*F’* value of 8.20 and 14.19, and *‘p’* value of 0.0264 and 0.0087 (< 0.05) respectively. These results clearly indicate that the percentage drug release is strongly dependent on all the selected independent variables. This equation infers that the judicious combination of HPMC K4M, okra gum and LBG is necessary [30] to control and sustain the drug release for 24 h. Table 3 shows the results of the analysis of variance (ANOVA), which was performed to identify insignificant factors.

**Table 3:** Summary of ANOVA table for dependent variables from simplex lattice design

**Source (Linear mixture)**	**Sum of squares**	**Degree of freedom**	**Mean square**	**‘*F’ *value**	**Probability *‘p’ *value**
Hardness	4.12	2	2.06	3.04	0.1370
f_lag_ time	12739.02	2	6369.51	16.63	0.0062*
1 h drug release	37.79	2	18.90	3.30	0.1218
2 h drug release	117.80	2	58.90	3.13	0.1315
8 h drug release	947.0	2	473.50	8.20	0.0264*
24 h drug release	432.98	2	216.49	14.19	0.0087*

*p < 0.05 indicate model terms are significant^*^.

**Figure 4:**
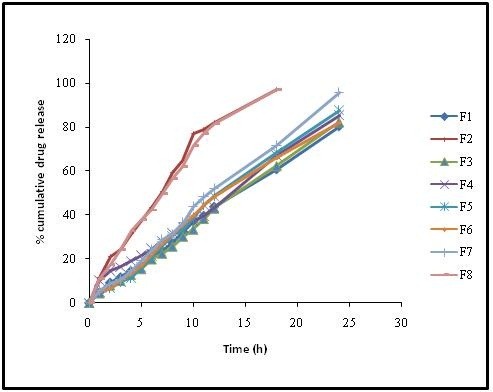
Comparative release profiles of ziprasidone HCl gastroretentive formulations.

**Figure 5:**
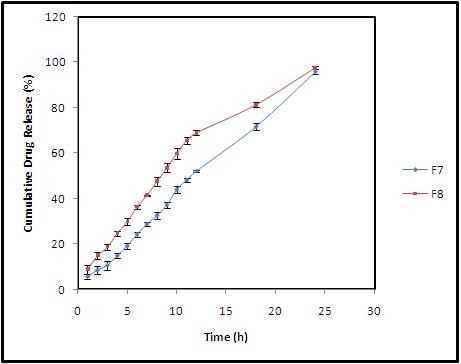
Comparative drug release profile of F7 and F8 formulation.

Based on this analysis, formulation F7 was arbitrarily selected as an optimized batch which releases the drug satisfactorily till the end of 24 h in spite of its high f_lag_ time of 43.33 ± 13.79 sec. In order to overcome the drawbacks of F7 formulation a checkpoint batch F8 prepared by considering the constraints and desirability to improve (Table 4) its *in vitro* performance. The experimental results of formulation F8 for f_lag_ time, total floating time and swelling index were found to be 37.33 ± 4.16sec, > 24 h and 204.0 ± 5.30% (up to 6 h) respectively. The *in vitro* drug release data was found to be sustained well compared to the most satisfactory formulation (F7) of 7 runs (Figure 4 and Figure 5). Predicted results were almost similar to the observed experimental values indicates the accuracy of the design (Table 5). All formulations were found to be buoyant for more than 24 h.

**Table 4:** Coded quantities of the check point batch “F8” and their desirability

**Constraints**						
**Name**	**Goal**	**Lower limit**	**Upper Limit**	**Lower weight**	**Upper weight**	**Importance**
HPMC K4M	Is in range	0	1	1	1	3
Okra gum	Is in range	0	1	1	1	3
LBG	Is in range	0	1	1	1	3
Floating lag time	Minimize	27	137	1	1	3
8h release	Minimize	25.69	59.39	1	1	3
24h release	Maximize	80.5	100	1	1	3
Solutions (Desirability 0.642)						
A		B	C	f_lag_ time (sec)	8h release	24 h release
0.946		-	0.054	33.66	51.26	98.45

**Table 5:** Comparison of experimented and predicted values of check point batch “F8”

**Parameter**	**Predicted values**	**Experimented values**
Hardness (kg/cm^2^)	5.46	5.68 ± 0.52
Floating lag time (sec)	33.66	37.33 ± 4.16
% Drug release at 8 h	51.26	47.53 ± 0.87
% Drug release at 24 h	98.45	97.58 ± 0.63

^a^All values are mean of 3 readings ± SD.

### Kinetic modeling on drug release profile

The release profile and kinetics of drug release are important because they correlate the *in vitro* and *in vivo* drug responses by comparing results of pharmacokinetics and dissolution profile patterns [31]. Hence, the cumulative drug release results of F7 and F8 formulation were fixed into various mathematical models and the results are shown in Table 6.

**Table 6:** Kinetic modeling of drug dissolution profiles

**Formulation code**	**Zero order**		**First order**		**Higuchi**		**Koresmeyar- peppas**	
	***r***^***2***^	***k***	***r***^***2***^	***k***	***r***^***2***^	***k***	***r***^***2***^	***n***
F7	0.993	4.089	0.878	0.056	0.948	23.50	0.982	0.954
F8	0.934	4.107	0.936	0.066	0.981	24.76	0.989	0.819

The drug release pattern of formulation (F7) was found to be highly linear, and close to infinity as indicated by their high regression value as r^2^ = 0.993. Therefore it was ascertained that the drug permeation from these formulation could follow either near zero or zero order kinetics.

The *in vitro* drug release pattern of F8 showed the highest regression value (r^2^ = 0.989) for Koresmeyar- peppas model. The ‘n’ value was found to be between 0.5 and 1 suggesting that release of drug follows anomalous (non-fickian) diffusion mechanism. Release kinetics may be following both diffusion and erosion mechanism from these natural gums [32].

#### *In vivo* floatability

**Figure 6:**
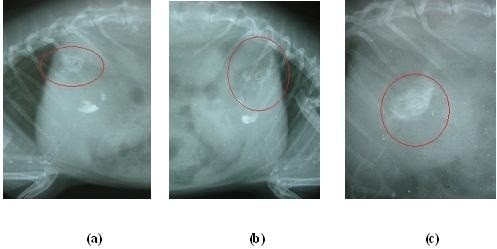
X-ray photographs showing floating ability of F8 formulation at different time interval (a) 6 h, (b) 12 h and (c) 24 h.

*In vivo* floatability studies conducted for F8 showed that the tablet formulation did not adhere to the gastric mucous and floated in the gastric fluid for more than 24h. To make the tablet X-ray opaque barium sulphate was incorporated into the tablet. The amount of barium sulphate (2mg per tablet) was low enough to enable the tablet to float, at the same time it was sufficient to ensure visibility by X-ray. This was evident by the X-ray photographs taken at 6 h, 12 h & 24 h (Figure 6).

## Conclusions

Ziprasidone HCl gastroretentive tablet is developed using naturally occurring plant based polymers showed desirable high-drug content, optimal hardness, floatability, swelling index and adequate release characteristics. The systematic formulation approach using simplex lattice design in the study helped in understanding the effect of formulation variables. The use of plant-based polymeric can be a good replacement for synthetic polymers in the development of controlled release dosage forms, because plant based materials can be modified to meet the requirements of drug delivery systems. Formulations prepared by such renewable and eco-friendly plant resources can be considered as promising SR polymers substances to bring about sustained release action, supported by more elaborated research in this aspect.

## Competing interests

The authors declare that they have no competing interest.

## Authors’ contributions

SB is involved in design of research protocol, statistical assessment of all the results and drafted the manuscript. AJ is participated in the development of formulation and *in vitro* evaluation of formulation. HN has collected and prepared the Okra gum for formulation use and carried out the *in vivo* floatability test of the formulation. All authors read and approved the final manuscript.
